# Subversion of Ras Small GTPases in Cutaneous Melanoma Aggressiveness

**DOI:** 10.3389/fcell.2020.575223

**Published:** 2020-09-23

**Authors:** Cheila Brito, Duarte C. Barral, Marta Pojo

**Affiliations:** ^1^Unidade de Investigação em Patobiologia Molecular (UIPM) do Instituto Português de Oncologia de Lisboa Francisco Gentil E.P.E., Lisbon, Portugal; ^2^CEDOC, Faculdade de Ciências Médicas, NOVA Medical School, Universidade NOVA de Lisboa, Lisbon, Portugal

**Keywords:** ras Small GTPases, biomarkers, metastasis, cancer progression, cancer therapies, cutaneous melanoma

## Abstract

The rising incidence and mortality rate associated with the metastatic ability of cutaneous melanoma represent a major public health concern. Cutaneous melanoma is one of the most invasive human cancers, but the molecular mechanisms are poorly understood. Moreover, currently available therapies are not efficient in avoiding melanoma lethality. In this context, new biomarkers of prognosis, metastasis, and response to therapy are necessary to better predict the disease outcome. Additionally, the knowledge about the molecular alterations and dysregulated pathways involved in melanoma metastasis may provide new therapeutic targets. Members of the Ras superfamily of small GTPases regulate various essential cellular activities, from signaling to membrane traffic and cytoskeleton dynamics. Therefore, it is not surprising that they are differentially expressed, and their functions subverted in several types of cancer, including melanoma. Indeed, Ras small GTPases were found to regulate melanoma progression and invasion. Hence, a better understanding of the mechanisms regulated by Ras small GTPases that are involved in melanoma tumorigenesis and progression may provide new therapeutic strategies to block these processes. Here, we review the current knowledge on the role of Ras small GTPases in melanoma aggressiveness and the molecular mechanisms involved. Furthermore, we summarize the known involvement of these proteins in melanoma metastasis and how these players influence the response to therapy.

## Introduction

Melanoma derives from the malignant transformation of melanocytes, which are melanin-producing cells located in the epidermis, eyes, meninges, esophagus, and mucous membranes (Ali et al., 2013). According to the site of origin of this malignancy, three main subtypes of melanoma are defined: cutaneous, uveal, and mucosal (Kuk et al., 2016). While cutaneous melanoma accounts for 90% of all melanoma cases, uveal and mucosal melanomas are relatively rare (Leonardi et al., 2018).

Cutaneous melanoma, herein referred to as melanoma, represents the most lethal skin neoplasm, leading to 60–75% of the mortality rate related to skin malignancies, even though it accounts for only 5% of all skin tumors (Bandarchi et al., 2010; Potrony et al., 2015; Esteva et al., 2017). Despite the efforts to prevent and detect melanoma early on, the incidence of this type of cancer has been increasing worldwide (Guy et al., 2015; Siegel et al., 2019). Presently, the staging of melanoma (I–IV) is based on tumor thickness, presence or absence of ulceration, lymph node involvement, and distant metastasis (Zbytek et al., 2008). Essentially, stage I and II melanoma show no evidence of regional or distant metastasis, in contrast to stage III and IV melanoma, which are characterized by lymph node and distant metastasis, respectively (Mohr et al., 2009). Early-stage melanoma is often associated with a favorable prognosis, with a 5-year survival rate of up to 90% (Luke et al., 2017; Allemani et al., 2018). On the other hand, stage IV melanoma is characterized by a 5-year survival rate of only 16% (Luke et al., 2017; Allemani et al., 2018; Cronin et al., 2018).

The most aggressive melanomas spread from the primary tumor site to surrounding tissues and frequently demonstrate a tendency to resist to available therapies (reviewed in Luís et al., 2020). Indeed, the high mortality rate of melanoma patients is mainly associated with its elevated metastatic ability (Zbytek et al., 2008). Currently, the treatment of advanced-stage melanoma is based on surgical excision, targeted therapies, and immunotherapies. BRAF and MEK inhibitors are targeted therapies approved by the FDA for the treatment of patients with *BRAF*-mutant melanomas (Grimaldi et al., 2017). The treatment of metastatic melanoma patients with highly selective BRAF-inhibitors improves both overall and progression-free survival (OS and PFS) (Sosman et al., 2012; Wong and Ribas, 2016; Domingues et al., 2018). However, only half of these patients demonstrate a positive response to targeted therapies and this response tends to be limited over time (Eroglu and Ribas, 2016).

The immunotherapies also available for advanced-stage melanoma are based on CTLA-4 and PD-1 blockers, which confer a survival benefit and more durable responses, compared to targeted therapies (Luke et al., 2017; O’Donnell et al., 2019; Yu et al., 2019). However, primary resistance occurs in 40–65 and 70% of metastatic melanoma patients submitted to anti-PD-1 and anti-CTLA-4 therapies, respectively. Moreover, from the initial responders, around 20–30% develop secondary resistance (Gide et al., 2018). Recently, it was shown in stage III melanoma patients that the neoadjuvant treatment with immunotherapies and targeted therapies is associated with higher rates of OS, disease, recurrence, and metastasis free-survival, resulting in a more efficient therapeutic approach to impair melanoma progression (Eggermont et al., 2016, 2018; Long et al., 2017; Weber et al., 2017; Maio et al., 2018; Song et al., 2019).

Despite the growing understanding of melanoma biology and the improvement in its treatment over the last decades, the genetic basis of melanoma metastasis is unclear (Liu and Sheikh, 2014). Consequently, the unveiling of signaling pathways and complex interactions contributing to melanoma progression could provide relevant knowledge for the development of novel and efficient therapies (Damsky et al., 2011).

Rat sarcoma (Ras) superfamily of small guanosine-5′-triphosphate (GTP)ases regulate many essential cellular activities such as cell signaling, membrane trafficking and cytoskeleton dynamics (Vetter and Wittinghofer, 2001; Zhen and Stenmark, 2015; Casalou et al., 2016, 2019; Toma-Fukai and Shimizu, 2019). Although Ras small GTPases control crucial physiological functions in cell homeostasis, several superfamily members are involved in the aberrant activation of signaling cascades that play a central role in a broad spectrum of human diseases, including cancer (Aspenström, 2018; Casalou et al., 2020; Gopal Krishnan et al., 2020). In recent years, a growing interest in the functions of small GTPases in the context of cancer has emerged. Considering the multiplicity of cellular processes in which these proteins are involved, it is essential to understand their usefulness as potential biomarkers and/or therapeutic targets. Notably, in melanoma, the dysregulated expression and/or activity of these proteins has been associated with cancer cell migration and invasion (Wong and Ribas, 2016; Liu W.N. et al., 2017; Liu et al., 2019; Wen et al., 2017; Huang et al., 2018). Therefore, the normal functions of small GTPases can be subverted by melanoma cells to spread and invade, leading to metastasis (Wong and Ribas, 2016; Huang et al., 2018). As such, melanoma, which is one of the most invasive types of cancer, is a suitable and useful model to explore the mechanisms underlying the roles of Ras superfamily members in cancer aggressiveness.

Here, we review the current evidence supporting the role of several Ras small GTPases in melanoma aggressiveness, progression, and response to therapy. Indeed, a considerable number of proteins from this superfamily are dysregulated in melanoma, mostly being over-activated and implicated in various molecular networks involved in melanoma growth, metastasis, and resistance to therapy.

## RAS Superfamily of Small Gtpases: Nomenclature and Regulation

Ras superfamily members regulate cytoskeleton remodeling, membrane trafficking, cell signaling, and nuclear transport, being essential for several cellular processes such as cell proliferation, differentiation and motility (Vetter and Wittinghofer, 2001; Zhen and Stenmark, 2015; Casalou et al., 2016, 2019; Toma-Fukai and Shimizu, 2019). Interestingly, Ras was the first small GTPase identified (Feinberg et al., 1983) and there are now more than 150 known members of this superfamily, based on their sequence homology and biochemical and functional similarities (Wennerberg et al., 2005; Rojas et al., 2012; Liu W.N. et al., 2017). The Ras superfamily comprises 5 families: Ras, Rho, Rab, Ran, and Arf (Rojas et al., 2012).

**FIGURE 1:**
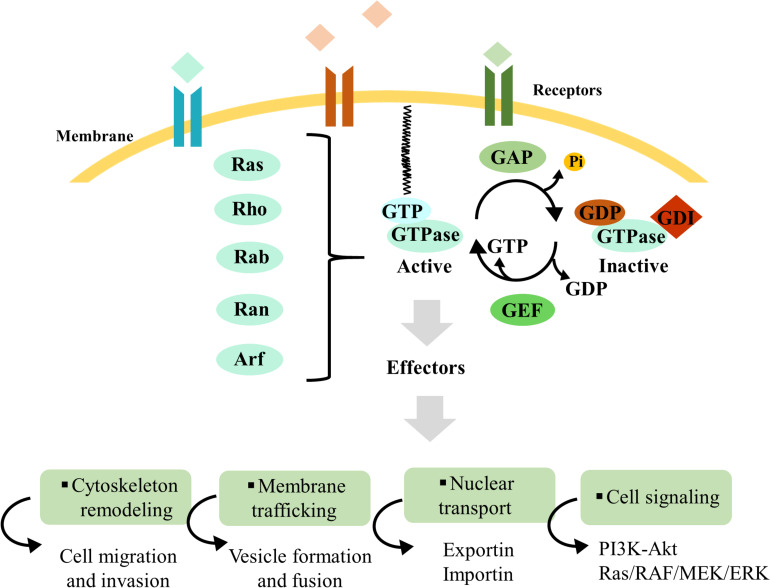
Overview of Ras superfamily small GTPase regulation. Ras superfamily protein regulation mechanisms and downstream interaction with effectors, which control cytoskeleton remodeling, membrane traffic, nuclear transport, and cell signaling. Guanine-nucleotide exchange factors (GEFs) and GTPase-activating proteins (GAPs) are pivotal regulators for small GTPase activation and inactivation, respectively. Besides these two regulators, guanosine nucleotide dissociation inhibitors (GDIs) are a distinct class of proteins that interact with small GTPases, preventing GDP dissociation and maintaining these proteins in their inactive form. Guanosine triphosphate (GTP)-bound and guanosine diphosphate (GDP)-bound GTPases are represented in blue and brown, respectively. The small black curved arrows represent the input and output of GDP and GTP during the cycles of GTPases activation and inactivation, as well as the release of the phosphate group. The specific names of the membrane and receptors are not mentioned, because these proteins can be present in distinct organelles.

A distinguishing feature of these proteins is that they alternate between an inactive GDP-bound and an active GTP-bound state (Cherfils and Zeghouf, 2013; Lin et al., 2018; Toma-Fukai and Shimizu, 2019). This molecular switch is mediated by GAPs, responsible for GTP hydrolysis to GDP, and GEFs, which catalyze the exchange of GDP to GTP (Bos et al., 2007) (Figure 1). Besides these two regulators, GDIs are a distinct class of proteins that interact with small GTPases, preventing GDP dissociation and maintaining these proteins in their inactive form (Rak et al., 2003). In general, active small GTPases localize to the plasma membrane or intracellular membranes; when these proteins are inactive, they are not membrane-bound and localize to the cytoplasm (Jackson and Bouvet, 2014; Kim et al., 2018; Goryachev and Leda, 2019) (Figure 1). GEFs, GAPs, and GDIs are essential regulators of small GTPase activity, allowing their spatiotemporal control and activation.

## RAS Family Members Involved in Melanoma Growth, Aggressiveness, and Response to Therapy

The Ras family was the first from the superfamily to be described, comprising approximately 36 members, divided into six subfamilies: Ras, Ral, Rap, Rad, Rheb, and Rit (Wennerberg et al., 2005; Goitre et al., 2014). Notably, the Ras subfamily includes 3 isoforms: Hras, Nras, and Kras, described several decades ago due to their oncogenic activation in several tumors (reviewed in Hobbs et al., 2016; Kano et al., 2016; Zhou et al., 2016). Previous studies demonstrated that 27% of all human cancers contain missense gain of function mutations in *RAS* genes (Prior et al., 2012; Hobbs et al., 2016).

**FIGURE 2:**
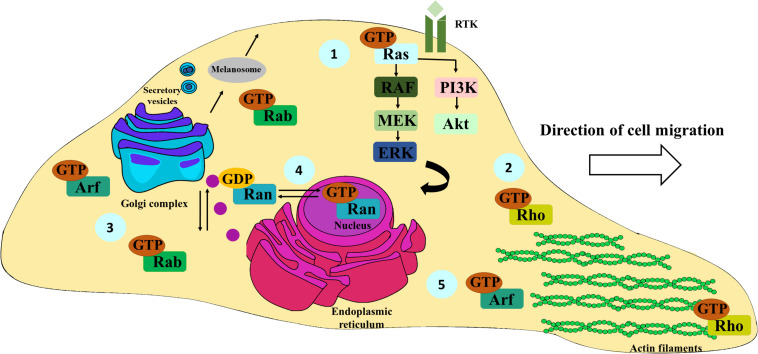
Schematic representation of the most important functions of Ras, Rho, Rab, Ran, and Arf family members. Ras proteins are mainly involved in the activation of the Ras/RAF/MEK/ERK and PI3K-Akt signaling pathways, fundamental for cell survival, proliferation, and differentiation; receptor tyrosine kinase (RTK) **(1)**. Rho family members are essentially associated with actin cytoskeleton remodeling, pivotal for cell migration and spreading **(2)**. Rab proteins are key in ensuring the specificity of vesicular transport. Particularly, they also have an important role in the formation, distribution, and secretion of melanosomes from melanocytes and melanoma cells to keratinocytes. **(3)**. Ran proteins regulate the transport of cargoes between nucleus and cytoplasm **(4)**. Arf family proteins are key regulators of membrane traffic, controlling vesicle budding, tethering, and actin cytoskeleton organization **(5)**.

Indeed, 28% of all melanoma cases have mutations in the *NRAS* gene, being after *BRAF* mutations the second most frequent oncogenic alteration in this type of cancer (Akbani et al., 2015). More than 80% of *NRAS* mutations occur in codon 61 and induce conformational changes in NRAS motifs, blocking GTP hydrolysis by GAPs and promoting the prevalence of GTP-bound NRAS (Akbani et al., 2015; Parker and Mattos, 2018). Consequently, the constitutive activation of these proteins triggers the aberrant activation of Ras/RAF/MEK/ERK and PI3K/Akt signal transduction pathways, which regulate cell proliferation, growth, differentiation, and survival (McCubrey et al., 2015; Santos and Crespo, 2018) (Figure 2). Additionally, *NRAS* upregulation was verified in melanoma samples, when compared to normal skin tissues (Liu S. et al., 2017). As expected, *NRAS* silencing in melanoma cell lines significantly reduces cell proliferation, migration, and invasion and promotes cell apoptosis (Table 1) (Liu S. et al., 2017). Consistently, *in vivo* experiments showed a decrease in melanoma growth after NRAS depletion, associated with the suppression of Ras/RAF/MEK/ERK and PI3K/Akt pathways (Liu S. et al., 2017). Therefore, the overactivation of these signaling pathways promotes the uncontrolled proliferation of melanoma cells, contributing to melanoma growth and aggressiveness (Yajima et al., 2012; Liu S. et al., 2017).

**TABLE 1:** Summary of the known roles of RAS superfamily small GTPases in cutaneous melanoma progression, resistance to therapy and clinical relevance.

**Family**	**Member name**	**Expression**	**Effect on cutaneous melanoma**	**References**
Ras	NRAS	↑	No consensus regarding prognostic value	Devitt et al., 2011; Jakob et al., 2012; Carlino et al., 2014; Sperduto et al., 2017
			Absent in benign lesions	Shain et al., 2015
			Increased proliferation, invasion, and migration	Yajima et al., 2012; Liu S. et al., 2017
			Thicker melanomas, elevated mitotic rate	Devitt et al., 2011; Ellerhorst et al., 2011; Heppt et al., 2017
	RAL	↑	Tumorigenic growth	Zipfel et al., 2010
			Malignant phenotypes	Mishra et al., 2010
	RAP1	↑	Melanoma growth and invasion	Hernández-Varas et al., 2011; Rodríguez et al., 2017
			Resistance to MAPK inhibitors	Zhou et al., 2020
Rho	CDC42	↑	Mesenchymal–amoeboid transition	Gadea et al., 2008
			Poor prognostic factor	Tucci et al., 2007
			Resistance to BRAF inhibitors	Mohapatra et al., 2019
	RAC1	↑	Increased melanoma thickness, mitotic rate and ulceration	Mar et al., 2014
			Melanoma growth and invasion	Revach et al., 2016; Mohan et al., 2019
			Promotes tumorigenesis	Lionarons et al., 2019
			Resistance to BRAF and MEK inhibitors	Watson et al., 2014; Revach et al., 2016; Mohan et al., 2019
			Modulates melanoma immunogenicity	Vu et al., 2015
	RHOA	↑	Regulates cell proliferation, migration, invasion	Guo et al., 2016; Wen et al., 2017; Liu et al., 2019
			UV protection of melanoma cells	Espinha et al., 2016
			Smaller tumors and absence of metastases	Kaczorowski et al., 2019
			Prognosis biomarker of prolonged OS	Kaczorowski et al., 2019
	RAB3A	N.A.	Melanosome transport and distribution	Araki et al., 2000
	RAB4A	N.A.	Production of pro-Cathepsin L	Barbarin and Frade, 2011
	RAB5A	↑	Melanoma migration, invasion, and metastasis	Silva et al., 2016
	RAB7	↑	Upregulated in earlier stages of tumorigenesis	Alonso-Curbelo et al., 2014
			Increased proliferative rate	Alonso-Curbelo et al., 2014
		↓	Low expression in more aggressive phenotypes	Alonso-Curbelo et al., 2014
			Expression pattern contributes for tumorigenesis and metastasis regulation	Alonso-Curbelo et al., 2014, 2015
Rab	RAB8	N.A.	Melanosome transport and distribution	Chakraborty et al., 2003
	RAB17	N.A.	Melanosome transport and distribution	Beaumont et al., 2011
			Melanoma growth	Gilot et al., 2017
	RAB22A	↑	Prognostic factor of poor outcome	Su et al., 2016
	RAB27A	↑	Melanosome distribution	Hume et al., 2001
			Regulates cell invasion and metastasis	Guo et al., 2019
			Prognostic biomarker of poor outcome	Guo et al., 2019
	RAB38	↑	Regulates cell invasion and metastasis	Huang et al., 2018
			Prognostic biomarker of poor outcome	Huang et al., 2018
Ran	RAN	↑	High levels in metastatic melanoma	Pathria et al., 2012; Caputo et al., 2015
Arf	ARF6	↑	Increased metastatic disease burden	Tague et al., 2004; Muralidharan-Chari et al., 2009; Hongu et al., 2016; Wong and Ribas, 2016
			Promotes lung colonization	Grossmann et al., 2013; Hongu et al., 2016; Wong and Ribas, 2016
			Early and late stages of melanoma progression	Grossmann et al., 2013; Wong and Ribas, 2016
			Promotes lymphangiogenesis	Lin et al., 2017

*↑, upregulated; ↓, downregulated; N.A., data not available.*

*NRAS* mutations are frequently detected in elderly melanoma patients, and they are preferentially found in the most aggressive histological subtype of melanoma, i.e., nodular melanoma, compared to the other histological subtypes (Lee et al., 2011; Hacker et al., 2013). Some authors reported that *NRAS* mutations are not prognostic factors for metastatic melanoma patients (Carlino et al., 2014; Sperduto et al., 2017). Both studies focused on a subset of metastatic melanoma patients with brain metastases and no MAPK inhibitor therapy, respectively (Carlino et al., 2014; Sperduto et al., 2017). Nevertheless, *NRAS* mutational status was described as a prognostic factor of shorter OS for stage IV non-uveal metastatic melanoma and primary invasive melanoma patients (Devitt et al., 2011; Jakob et al., 2012). The differences found between these studies could be explained by sampling size (varying between 193 and 823 patients) and by the subset of patients included in each study (Devitt et al., 2011; Jakob et al., 2012; Carlino et al., 2014; Sperduto et al., 2017). In this context, it would be important to clarify the role of these mutations in the prognosis of melanoma patients by staging to understand whether *NRAS* mutations could be important biomarkers to predict the behavior of this disease. Additionally, *NRAS* mutations were categorized as a type of mutation characteristic of intermediate skin lesions (Shain et al., 2015). In contrast, in benign lesions, these alterations are absent, suggesting their essential role during melanoma progression (Table 1) (Shain et al., 2015). Presently, there is evidence that *NRAS* mutations are associated with thicker melanomas, elevated mitotic rates, and a higher propensity for lymph node metastasis, highlighting the importance of these alterations in the clinical setting (Devitt et al., 2011; Ellerhorst et al., 2011; Heppt et al., 2017). Thus, *NRAS* mutational analysis should be included in melanoma routine diagnosis, given its relevance as a biomarker of melanoma aggressiveness and easy evaluation.

Considering that *NRAS* mutations are present in almost one third of melanoma cases, a successful targeted therapy would be expected by now. However, despite the enthusiasm, the therapeutic strategies developed so far have been mainly focused on KRAS and no effective NRAS targeted therapies have been approved (Kirchberger et al., 2018). Interestingly, MEK inhibitors have demonstrated a modest improvement in the PFS of *NRAS*-mutant melanoma patients even when compared with chemotherapy with dacarbazine (Ascierto et al., 2013; Shain et al., 2015; Kirchberger et al., 2018). MEK-targeted therapies involve cell-cycle arrest, which is in agreement with the *in vivo* studies showing melanoma growth suppression in mice treated with MEK inhibitors (Vogel et al., 2015). Moreover, according to one study, patients with *NRAS*-mutant melanomas would benefit from the administration of anti-CTLA-4 and anti-PD-1 therapies, since they demonstrated a better response to any kind of immunotherapy and an improved PFS, compared to patients with wild-type melanomas or *BRAF*-mutant melanomas (Johnson et al., 2015). Additionally, this response tends to be more stable and durable in *NRAS*-mutant melanoma patients, probably due to the higher levels of PD-L1 in these tumors (Johnson et al., 2015). This finding reinforces the idea that *NRAS* mutational analysis should be included in routine diagnosis as a predictive biomarker of response to therapy, to select the group of patients that would benefit more from the administration of immunotherapies, even though specific therapeutic options do not exist for *NRAS-*mutant melanoma patients.

Undoubtedly, Nras is the GTPase belonging to the Ras superfamily with the most significant impact in melanoma. However, Ral and Rap1 also play an important role in the context of melanoma. RAL was described as an inducer of proliferation in melanoma cell lines, even in the presence of *NRAS* and/or *BRAF* oncogenic mutations (Mishra et al., 2010; Zipfel et al., 2010) (Table 1). Likewise, RAP1 is upregulated in melanoma cell lines and mediates melanoma cell proliferation and invasion, contributing to resistance to MAPK inhibitors (Hernández-Varas et al., 2011; Rodríguez et al., 2017; Zhou et al., 2020) (Table 1). Moreover, RAP1 inhibition reduces melanoma cell adhesion and migration by suppressing tumor cell extravasation and consequently lung colonization in *in vivo* models (Freeman et al., 2010). However, the molecular mechanisms by which RAL and RAP1 influence cell proliferation and migration and their respective prognostic value in melanoma patients have not yet been explored. Overall, the imminent role of *NRAS* mutations in melanoma aggressiveness and response to therapy has been well established, although much is still to discover about the impact of the remaining Ras family members in melanoma pathophysiology and as putative therapeutic targets.

## Rho Family Members Involved in Melanoma Migration and Invasion

The Rho family was the first to be associated with cell migration (Hall, 2012). Indeed, the approximately 20 known members of this family were found to be involved in actin cytoskeleton dynamics, membrane traffic, cell polarity and tissue morphogenesis (Figure 2) (Jaffe and Hall, 2005; Heasman and Ridley, 2008; Sadok and Marshall, 2014; Ridley, 2015). The structural architecture and the continuous cytoskeleton remodeling are crucial for cell motility and adaptation to different microenvironments (Sadok and Marshall, 2014).

In the last decades, the role of Cdc42, Rac, and RhoA, the best-characterized members of this family, was extensively explored in relation to melanoma cell motility (Haga and Ridley, 2016). Specifically, these proteins are known to be involved in two mechanisms of melanoma cell migration, the amoeboid and mesenchymal types, which are interconvertible (Friedl and Wolf, 2003). Amoeboid migration is the preferential motility type shown by cells when they traverse the extracellular matrix. During this process, cells display a round shape (Sahai and Marshall, 2003). Moreover, it is mediated by RhoA and ROCK, which induce actomyosin contractility, essential for cell locomotory force against the microenvironment (Sahai and Marshall, 2003; Wilkinson et al., 2005). In contrast, cells present an elongated morphology during mesenchymal migration, which is a Rac-dependent and RhoA-independent process (Sahai and Marshall, 2003; Stengel and Zheng, 2011). Furthermore, the expression of Cdc42 constitutively active mutants induces cells to adopt a rounded shape increasing melanoma cell invasion and consequently allowing a mesenchymal–amoeboid transition. In contrast, Cdc42 depletion and expression of dominant negative mutants impair mesenchymal migration (Gadea et al., 2008) (Table 1). As such, Cdc42 is involved not only in amoeboid migration regulation but also in controlling mesenchymal migration. Nevertheless, the mechanisms involved are not known (Gadea et al., 2008). As melanoma cells can alternate between both types of migration in order to adapt to microenvironment changes (Friedl and Wolf, 2003), the targeting of both migration modes is likely to be required to block melanoma metastasis (Gadea et al., 2008).

Furthermore, CDC42 expression is upregulated in nodular melanoma patients who died from metastatic disease, compared to disease-free patients (Tucci et al., 2007). Additionally, a positive association between CDC42 expression and thickness was verified in metastatic melanoma patients (Tucci et al., 2007). Accordingly, CDC42 expression was suggested as a prognostic marker of shorter OS in melanoma patients (Tucci et al., 2007; Stengel and Zheng, 2011). Interestingly, a recent study showed that melanoma cell lines resistant to a BRAF inhibitor (PLX4032) display increased CDC42 activity compared to non-resistant melanoma cells (Mohapatra et al., 2019) (Table 1). As such, pharmacological drugs capable of interfering with CDC42 activity could induce conformational alterations in the actin cytoskeleton, impairing the invasiveness of BRAF-resistant melanoma cells (Mohapatra et al., 2019).

Despite mutations in Rho GTPase-encoding genes representing uncommon events, it was described that *RAC1*-activating mutations are present in approximately 4–9% of melanomas (Krauthammer et al., 2012; Araiza-Olivera et al., 2018; del Maldonado and Dharmawardhane, 2018). Particularly, the P29S substitution is the most common *RAC1* mutation found in malignant melanomas, inducing RAC1 spontaneous activation through the constitutive GDP/GTP nucleotide exchange (Hodis et al., 2012; Davis et al., 2013). From a clinical point of view, RAC1^P29S^ mutation is associated with increased melanoma thickness, high mitotic rate, ulceration, and the occurrence of the nodular melanoma subtype (Mar et al., 2014) (Table 1). Remarkably, the increased mitotic rate observed is due to the formation of lamellipodia, which are dependent on continuous actin polymerization and activate proliferative signaling cascades by inhibiting the neurofibromatosis type 2 (NF2)/Merlin tumor suppressor (Mohan et al., 2019). Consequently, NF2/Merlin inhibition by phosphorylation of the Rac effector PAK promotes melanoma cell resistance to MAPK inhibitors (Mohan et al., 2019) (Table 1). Consistently, the ectopic expression of RAC1^P29S^ in melanoma cell lines increases resistance to BRAF and MEK inhibitor therapies (Van Allen et al., 2014; Watson et al., 2014; Araiza-Olivera et al., 2018). Indeed, using a mouse xenograft model, the authors observed that this mutation enhances melanoma growth and invasion and confers drug resistance against BRAF and MEK kinase inhibitors (Revach et al., 2016; Mohan et al., 2019) (Table 1). More recently, using transgenic mouse models, it was also demonstrated that Rac1^P29S^ can cooperate with BRAF^V600E^ to promote melanoma tumorigenesis (Lionarons et al., 2019). Besides triggering molecular cascades that are pivotal for melanoma aggressiveness and response to therapy, RAC1^P29S^ can also alter the immune background of these tumors. Accordingly, an increased PD-L1 expression was found in patients with RAC1^P29S^ mutant melanomas, compared with wild-type RAC1 melanoma patients (Vu et al., 2015) (Table 1). Hence, these studies suggest that the P29S mutation can modulate melanoma immunogenicity to suppress the immune response against the tumor. Considering the impact of RAC1^P29S^ mutation in melanoma, it would be interesting to assess its relevance as a predictive biomarker of response to anti-PD-1 therapies, since it could select the patients who would most benefit from this therapy (Vu et al., 2015).

In addition to CDC42 and RAC1 GTPases, RHOA is also part of molecular networks crucial for the control of melanoma proliferation, migration, and invasion (Wen et al., 2017). Indeed, it was described that CREPT promotes melanoma progression via RHOA upregulation and activation (Liu et al., 2019). Likewise, the inhibition of the RHOA GEF-H1 by a microRNA (miR-194) suppresses RHOA activation and consequently melanoma cell proliferation and invasion (Guo et al., 2016).

Recently, the role of RHOA in the effects caused by UV radiation in metastatic melanoma cells was assessed. For that purpose, RHOA was constitutively expressed or silenced in melanoma cells and subsequently the cells were exposed to UV. RHOA silencing was found to induce an increase in melanoma sensitivity to UV, increasing the number of DNA damages caused and dramatically reducing melanoma cell motility and invasion (Espinha et al., 2016). As such, RHOA can regulate DNA repair mechanisms, playing a central role in protection against UV radiation, by mediating genomic stability of melanoma cells (Espinha et al., 2016) (Table 1). Hence, this protein was suggested as a promising therapeutic target to sensitize melanoma cells to genotoxic damages (Espinha et al., 2016). Nevertheless, in the clinical context, immunohistochemical analyses showed that there is an increase in RHOA expression associated with smaller tumors and an increase of tumor-infiltrating lymphocytes (Kaczorowski et al., 2019) (Table 1). Furthermore, RHOA expression is also correlated with the absence of metastases and prolonged OS of melanoma patients (Kaczorowski et al., 2019). In contrast to other published studies (Guo et al., 2016; Wen et al., 2017), Kaczorowski et al. (2019) showed that RHOA might have an antitumoral activity in primary melanomas. Despite the reduced number of samples (*n* = 134), this is the first published study to analyze RHOA expression in patient-derived melanoma samples. Thus, the contradictory results could be explained by the distinct methodological approaches and melanoma models used, and the absence of a clear distinction between the impact of RHOA expression on primary vs. metastatic melanoma. Additionally, the results obtained could also be influenced by the detection of other isoforms of Rho GTPases. Thus, further studies employing murine models and using larger cohorts of patients should be performed to clarify the impact of RHOA expression in melanoma aggressiveness and progression.

Altogether, the studies described demonstrate that cytoskeleton dynamics, controlled by Rho GTPases, plays a critical role in cell signaling, which consequently dictates the mechanisms of melanoma cell proliferation and motility. Hence, cell morphology can be a key factor to promoting melanoma cell growth and metastasis. Clinically, CDC42, RAC1, and RHOA expression in patient samples can provide relevant knowledge about melanoma thickness, ulceration, prognosis, and drug resistance. Similar to RAS family proteins, the interest in targeting RHO GTPases for melanoma patients treatment is increasing, although few drugs passed beyond an earlier preclinical stage (Lin and Zheng, 2015).

## Rab Family Proteins and Their Role in Melanoma Metastasis and Microenvironment Modulation

Rab GTPases represent the largest evolutionary conserved family within the Ras superfamily, containing almost 70 members described in humans (Wandinger-Ness and Zerial, 2014; Guadagno and Progida, 2019). Early studies demonstrated that these proteins are present in almost all cell compartments and ensure the specificity and directionality of membrane traffic (Stenmark and Olkkonen, 2001; Zhen and Stenmark, 2015) (Figure 2). Indeed, Rab proteins are known to regulate all steps of membrane traffic, namely, vesicle formation, motility, tethering, docking, and fusion (Bhuin and Roy, 2014) (Figure 2).

Rab proteins have been one of the families of the Ras superfamily mostly studied (Guadagno and Progida, 2019). However, their role in tumor progression emerged more recently. A broad study using a directed proteomic quantification approach evaluated the differential expression of Ras small GTPases in primary and metastatic melanoma cell lines, in order to establish a general profile of the most relevant ones (Huang et al., 2018). Based on this, RAB27A and RAB38 were found upregulated in metastatic melanoma, relative to the matched primary melanoma cell lines derived from human samples (Huang et al., 2018).

The localization of Rab27a to melanosomes and its role in controlling the distribution of these melanin-containing compartments in melanoma cells was described almost two decades ago (Hume et al., 2001). Both Rab27a and Rab38 promote cell invasion, although the mechanisms used by each one are distinct. On the one hand, RAB27A induces melanoma invasion through the production of pro-invasive exosomes carrying out protein cargos such as EPHB4, Glypican1, and BMP 1, previously described as a pivotal protein for melanoma cell motility (Yang et al., 2006; Aikawa et al., 2008; Peinado et al., 2012; Guo et al., 2019, 2020). On the other hand, RAB38 regulates melanoma cell invasion by increasing the expression and activity of two MMPs, MMP-2 and -9 (Huang et al., 2018). This effect is also mediated by the MITF, the master regulator of melanocyte development, which leads to an increase in RAB38 expression in metastatic melanoma (Huang et al., 2018). Considering the role of these proteins in melanoma invasion and the observation that *RAB27A* and *RAB38* expression were associated with shorter OS of melanoma patients, these GTPases were proposed as drivers of melanoma metastasis (Huang et al., 2018; Guo et al., 2019) (Table 1). Similarly, RAB22A expression is also a prognostic factor of poor outcome in melanoma patients, showing an increased expression in primary melanomas compared with benign nevi (Su et al., 2016; Zhou et al., 2017). Notably, this protein was associated with the proliferation, migration, and invasion ability of melanoma cells, being proposed as a potential therapeutic target in melanoma (Table 1).

Additionally, RAB7 has a relevant role not only in the earlier stages of melanoma, regulating melanoma cell proliferation, but also in the late stages of this disease (Alonso-Curbelo et al., 2014; Zhao et al., 2017) (Table 1). Interestingly, RAB7 preferentially accumulates in melanoma cell lines and samples compared to other types of cancer, as part of a lysosomal-associated signature (Alonso-Curbelo et al., 2014, 2015). Indeed, RAB7 regulates lysosomal-associated proteolytic activity in melanoma cell lines. However, immunohistochemical analysis of normal skin and primary and metastatic melanoma tissues derived from patients revealed that RAB7 expression levels are not constant during melanoma progression (Zhao et al., 2017). In earlier stages of melanoma development, this protein is upregulated (Zhao et al., 2017). In contrast, when melanoma cells start to acquire a more aggressive phenotype displaying invasive features, RAB7 is found downregulated (Zhao et al., 2017). Therefore, low levels of RAB7 expression in primary tumors are associated with an increased risk of melanoma metastasis (Alonso-Curbelo et al., 2014). The discovery of this melanoma cell-specific mechanism suggests that RAB7 expression pattern can be important to understand the mechanisms of melanoma aggressiveness and metastasis (Table 1).

In addition to Rab27a, other Rab proteins such as Rab3a, Rab8, and Rab17 are involved in melanosome transport and distribution (Figure 2), including in melanoma cells, playing an important function in the transfer of melanin from melanocyte dendrites to keratinocytes (Araki et al., 2000; Chakraborty et al., 2003; Beaumont et al., 2011) (Table 1). Despite being involved in melanosome transport, RAB17 also enhances melanoma growth *in vivo* (Gilot et al., 2017) (Table 1). Indeed, Rab proteins not only are involved in several molecular mechanisms subverted by cancer cells but also contribute for tumor microenvironment modification, conferring appropriate conditions for melanoma progression. For example, RAB4A expression directly affects the production of pro-cathepsin L, a cysteine protease that contributes for the resistance to complement-mediated cell lysis in melanoma cells (Barbarin and Frade, 2011) (Table 1). Hence, RAB4A upregulation promotes an increase in pro-cathepsin L secretion, although the mechanisms by which this occurs are unknown. Moreover, under hypoxia conditions, Rab5a expression is upregulated and seems to be required for melanoma migration, invasion, and metastasis *in vivo* (Silva et al., 2016), although its prognosis value was not determined (Table 1).

Several studies have reported the dysregulation of Rab proteins in melanoma, although few have yet elucidated the molecular networks involved, which could be important to identify new potential therapeutic targets (Huang et al., 2018). It is widely accepted that exosomes are mediators of cell–cell communication and the ones derived from tumor cells can carry factors that induce malignant transformation (Maia et al., 2018). Since the formation and delivery of exosomes is mediated by Rab proteins, it is crucial to test their association with metastasis molecular signatures to uncover effective biomarkers of melanoma progression. In the clinical setting, these studies would be of extreme relevance to understand whether there is some correlation between the expression of RAB proteins and melanoma thickness, ulceration, and metastasis.

## Differential Expression of RAN Family Proteins During Melanoma Progression

The Ran family is composed by one single member in humans, which is the most abundant small GTPase and is mainly found in the interphase nucleus (Zheng, 2004; Wennerberg et al., 2005). In contrast to the other families, Ran GTPase is specialized in the transport of cargoes between the cytoplasm and the nucleus through the nuclear pore complex (Güttler and Görlich, 2011) (Figure 2). The communication between these two cellular compartments promotes the import of transcriptional factors that are essential for genome transcription (Matchett et al., 2014). Inside the nucleus, Ran GTPase is predominantly present in the GTP-bound form, being essential to maintain the exportin–cargo interaction, while in the cytoplasm, Ran is mainly found in the GDP-bound form, required for the cargo release (Cavazza and Vernos, 2016). The switch from GTP-bound to GDP-bound Ran is required for the export of macromolecules to the cytoplasm, by stabilizing the complex formed between the exportin and the bound cargo (Kuersten et al., 2001). In the cytoplasm, where GTP-bound Ran is inactivated, the complexes formed by the exportin and the cargoes are disassembled in order to release the cargo (Dahlberg and Lund, 1998). In contrast, GDP-bound Ran is required in the cytoplasm for nuclear cargo import and during this process GTP-bound Ran is only needed for the final release of the imported cargo (Lui, 2009). Besides controlling cytoplasm-nucleus trafficking, Ran also regulates mitotic spindle assembly during mitosis (Zheng, 2004).

According to a study by Caputo et al. (2015) increased RAN expression levels were detected in 48% of metastatic melanoma patients (Table 1). Additionally, RAN upregulation was also found in melanoma cell models, compared to normal melanocytes and in metastatic melanoma, compared to primary melanoma samples and melanocytic nevi (Pathria et al., 2012; Caputo et al., 2015). Moreover, it was described that AurkA could be a downstream target of Ran, as Ran silencing reduces AurkA expression (Caputo et al., 2015). Since Ran regulates mitotic spindle assembly, its downregulation may generate genomic instability and decrease melanoma cell proliferation, as previously verified for ovarian and pancreatic cancer cell lines (Barrès et al., 2010; Deng et al., 2013). Nevertheless, the role of this protein in melanoma proliferation and invasion was not explored yet. Indeed, the nucleocytoplasmic transport of several molecules could be enhanced by Ran upregulation, increasing the cytoplasmic accumulation of several proteins pivotal for cell proliferation and invasion. As such, further studies should be performed to evaluate whether Ran could be an important player during melanoma tumorigenesis and progression.

## ARF Family Members Control Melanoma Metastasis Through the Activation of Several Molecular Networks

Similar to the other members of the Ras superfamily, Arf family proteins are key regulators of membrane traffic, controlling vesicle budding, tethering, and actin cytoskeleton organization (Chavrier and Goud, 1999; D’Souza-Schorey and Chavrier, 2006) (Figure 2). This family includes 6 members (Arf1–6; Arf2 is not expressed in humans), which are classified into three classes according to their sequence homology: class I (Arf1 and Arf3), class II (Arf4 and Arf5), and class III (Arf6) (Donaldson and Jackson, 2011). Based on their structural similarity, Arl proteins are also included in the Arf family (Kahn et al., 2006). Overall, this family includes a total of 30 members in humans (Sztul et al., 2019).

Currently, among Arfs, only Arf6 was reported as playing a critical role in melanoma. Despite the difficulties related with the measurement of Arf6 activation state due to GTP instability, the aberrant activation of this protein was confirmed in human melanoma samples compared with matched normal skin tissue (Wong and Ribas, 2016). Additionally, *ARF6* ectopic expression reduces tumor growth and increases the invasive ability of melanoma cells in an immunocompromised mouse model (Muralidharan-Chari et al., 2009). Similarly, *Arf6* overexpression increases metastatic disease burden, accelerating melanoma metastasis and lung colonization in an immunocompetent mouse model (Hongu et al., 2016; Wong and Ribas, 2016) (Table 1). Indeed, Arf6 can modulate distinct molecular networks essential for early and late stages of melanoma metastasis (Grossmann et al., 2013; Wong and Ribas, 2016). Particularly, Arf6 role on melanoma progression and tumorigenesis is mediated by Ras/RAF/MEK/ERK signaling pathway, which activates Rac1, leading to cytoskeleton remodeling and the formation of invadopodia (Tague et al., 2004; Muralidharan-Chari et al., 2009). In addition to Ras/RAF/MEK/ERK, the PI3K-Akt pathway is also required for Arf6-mediated metastasis. Moreover, Arf6 was described to increase PI3K protein levels and be sufficient and indispensable for PI3K/Akt activation, kinases that are mainly located in peripheral cellular compartments and induce melanoma invasion (Wong and Ribas, 2016). Furthermore, WNT5A has emerged as an important player of cancer invasion. As expected, WNT5A activates ARF6 also in melanoma, controlling the shuttling of β-catenin between the plasma membrane and cytoplasm (Grossmann et al., 2013). In this context, ARF6 disrupts N-cadherin and β-catenin complexes, weakening adherens junctions, in order to regulate melanoma invasiveness and promote pulmonary metastasis (Grossmann et al., 2013). The role of Arf6 in several pathways related to melanoma dissemination has been extensively explored, and there is evidence that this protein also contributes for the lymphangiogenesis process occurring under physiological and pathological conditions (Lin et al., 2017). This effect is mediated by β1-integrin internalization, which is pivotal for the VEGF-C-associated cell migration and consequently for vascular network formation in melanoma (Lin et al., 2017) (Table 1).

In contrast with Arf proteins, the biochemical characterization and knowledge about the functions of Arls is lagging behind. Previously, the presence of a specific ARL1 variant (C148R) was assessed in 351 familial and sporadic melanomas and associated with an increased risk for heterozygous carriers to develop melanoma (Frank et al., 2006). To our knowledge, this is the only study describing the role of an Arl protein in melanoma. Considering the essential functions of Arls in membrane traffic and their impact in the tumorigenesis and progression of several types of cancer, it is likely that they play important roles in melanoma tumorigenesis and progression. Therefore, further studies are warranted to evaluate the role of Arl proteins in melanoma and the molecular pathways involved.

## Targeting Ras Small Gtpases

In the last decade, several inhibitors have been proposed to target Ras GTPases as an attempt to reduce cell migration and invasion, thereby impairing tumor progression (Prieto-Dominguez et al., 2019). Indeed, Ras small GTPases have been suggested as good candidate therapeutic targets, although to date there are no drugs currently available in the clinical practice that target these proteins (del Maldonado and Dharmawardhane, 2018; Prieto-Dominguez et al., 2019). The first therapeutic strategies designed for this purpose were aimed to inhibit their expression. However, Ras GTPases have been referred to as undruggable targets due to their structural features with limited small-molecule binding pockets (O’Bryan, 2019). Additionally, one of the main barriers to the success of these approaches is the ubiquitous expression of most of these proteins among human tissues and their essential physiological functions for cell homeostasis and survival (Casalou et al., 2020). Hence, the targeting of Ras GTPases could have harmful effects on tissues not affected by the disease. In this context, a growing interest in blocking GEF and GAP activities, inhibiting GTP or membrane binding, as well as in targeting of downstream effectors of these proteins is underway. Indeed, this strategy could overcome the damaging effects caused by the direct inhibition of proteins that play essential functions in most or all the cells in the body.

For instance, salirasib is a Ras inhibitor that blocks the membrane association of Ras proteins, whose safety and tolerability were already assessed in patients with refractory/relapsed tumors in a phase I trial (Furuse et al., 2018). This drug was indicated for phase II trials and seems to be a promising therapeutic strategy for patients with *RAS* mutations who have no specific therapeutic options (Furuse et al., 2018). EHT1864 is an inhibitor with high affinity for Rac GTPases, impairing their activity by inhibiting GTP binding (Katz et al., 2012). This inhibitor suppresses cell invasion and proliferation in triple-negative breast cancer cell lines by reducing the PI3K-Akt pathway activation (Hampsch et al., 2017). NSC23766 is another Rac1 inhibitor that blocks the interaction of Rac1 with its GEFs TRIO and TIAM1. Despite several *in vitro* assays having demonstrated a reduction in the invasion ability of distinct types of tumor cells after NSC23766 treatment, this inhibitor has been described as inefficient for clinical administration due to its low efficacy (Bid et al., 2013). LM11, a specific inhibitor of GEF binding to Arf1, reduces cell proliferation, migration, and metastasis in a zebrafish model of breast cancer (Xie et al., 2016). Rab7 was the first member of the Rab GTPase family to be the target of an inhibitor, namely, CID1067700 (Agola et al., 2012). Moreover, Rab25 is a target of the RFP14 peptide, which impairs Rab25 binding to GTP (Mitra et al., 2017). The design of small molecules targeting Rab GTPases is delayed compared with the discovery of inhibitors for other Ras GTPase families. The specificity of the inhibitors is of extreme importance to prevent the inhibition of other proteins that are not dysregulated.

Despite the difficulties associated with the design of efficient inhibitors for Ras GTPases, advances in the knowledge of the mechanisms involved in the function of these proteins could shed light on new promising therapeutic approaches.

## Conclusion and Future Perspectives

The Ras superfamily members play distinct and pivotal roles during melanoma tumorigenesis and progression (Table 1). Specifically, they are associated with melanoma patients’ response to targeted and immunotherapies, reinforcing their importance as potential predictive biomarkers. Rho GTPases play a prevalent function in melanoma metastatic processes, being fundamental to determining the mechanisms underlying amoeboid and mesenchymal types of migration. Rab proteins can mediate a vast number of invasion mechanisms, although it would be interesting to further assess their role in melanoma microenvironment modification, since there is evidence supporting the influence of these proteins in metabolic pathways to promote resistance to immune responses. Unfortunately, the current knowledge about the action of Ran and Arf family members in melanoma is limited, specifically in the case of Arl proteins, whose involvement in melanoma oncobiology remains unknown.

The metabolic heterogeneity of melanoma cells is another important factor contributing for their ability to adapt to the microenvironment conditions and induce tumor progression and metastasis. In this perspective, it would be interesting to investigate the mechanisms involved in the interplay between Ras small GTPases, tumor microenvironment, and metabolic pathways. Even more important than understanding the individual role of these proteins is to explore their cooperative contribution for complex metabolic–molecular–immune networks, given that compensatory mechanisms could mask their individual contribution. Considering the invasive properties of melanoma, Ras small GTPases could be important mediators to elucidate what patients show more propensity to develop a worse prognosis, defining the subset of patients requiring adjuvant therapy to prevent metastatic disease.

The use of Ras superfamily members as therapeutic targets may be an efficient approach for metastatic melanoma patients since they are master regulators of melanoma cell migration and invasion. Indeed, targeting the main pathways involved in melanoma metastasis could be the most promising strategy to decrease the aggressiveness and lethality of this type of neoplasm. Thus, the study of regulators and effectors that interact with Ras small GTPases should be a focus of research to develop therapeutic strategies that ensure the specific targeting of tumor cells. Furthermore, this specificity can also be achieved by resorting to drug delivery systems that only target the tumor.

Considering the complexity of the Ras superfamily members functions, much is still to discover about the singular and cooperative effects, as well as the spatiotemporal regulation of its members in melanoma. The understanding of melanoma biology will allow us to gain insight about the mechanisms involved in its progression and simultaneously on how to impair these processes. Overall, further studies are required to develop efficient and specific strategies to target these proteins, as an attempt to improve melanoma patients’ OS and life quality.

## Author Contributions

All authors listed have made a substantial, direct and intellectual contribution to the work, and approved it for publication.

## Conflict of Interest

The authors declare that the research was conducted in the absence of any commercial or financial relationships that could be construed as a potential conflict of interest.

## Abbreviations

Arf: ADP-ribosylation factor
Arl: Arf-like
Akt: protein kinase B
AurkA: Aurora kinase
BMP: bone morphogenetic protein
BRAF: V-raf murine sarcoma viral oncogene homolog B1
CREPT: cell-cycle-related and expression-elevated protein in tumor
CTLA-4: cytotoxic T-lymphocyte -associated antigen 4
EPHB4: ephrin type-B receptor 4
ERK: extracellular-signal-regulated kinase
FDA: Food and Drug Administration
GAP: GTPase-activating proteins
GDI: guanosine nucleotide dissociation inhibitors
GDP: guanosine diphosphate
GEF: guanine nucleotide exchange factors
GTP: guanosine-5 ′ -triphosphate
Hras: Harvey rat sarcoma
Kras: Kirsten rat sarcoma
MEK: mitogen-activated protein kinase
MITF: microphthalmia-associated transcription factor
MMP: metalloproteinases
NF2: neurofibromatosis type 2
Nras: neuroblastoma rat sarcoma
OS: overall survival
PAK: P21-activated kinase
PD-1: programmed cell-death protein 1
PFS: progression free survival
PI3K: phosphatidylinositol 3-kinase
RAF: rapidly accelerated fibrosarcoma
Rho: Ras homologous
Ran: Ras-like nuclear
Rab: Ras-like proteins in brain
Ras: Rat Sarcoma
ROCK: RHO kinase
UV: ultraviolet
VEGF-C: vascular endothelial growth factor-C.
